# Review of Diagnostic Imaging Modalities for the Surveillance of Melanoma Patients

**DOI:** 10.1155/2012/941921

**Published:** 2011-08-23

**Authors:** Yan Xing, Kate D. Cromwell, Janice N. Cormier

**Affiliations:** Department of Surgical Oncology, The University of Texas MD Anderson Cancer Center, Houston, TX 77030-4009, USA

## Abstract

As melanoma survival rates continue to increase, optimal surveillance strategies for recurrences are needed, as are effective imaging modalities. Therefore, we performed a meta-analysis to evaluate the current state of imaging modalities for surveillance of melanoma in the published medical literature to determine the sensitivity, specificity, and positive predictive values of ultrasonography, computed tomography (CT), positron emission tomography (PET), and CT-PET combined. Ultrasonography was found to be the most sensitive and specific for detecting lymph node metastases, and PET-CT was the most sensitive and specific for detecting distant metastases. In addition to identifying appropriate surveillance methods, future studies should focus on the most effective and cost-effective intervals for performing these tests. In addition, the results from the meta-analysis related to sensitivity and specificity of the tests should be made available to doctors in community practice.

## 1. Introduction

The number of melanoma survivors in the United States continues to increase, largely because of the successful treatment of newly diagnosed, early-stage disease [1]. However, up to 50% of these patients are at risk for recurrence, which is most common in the years immediately after diagnosis [2–7]. An estimated 20% of all first recurrences occur locally, 50% occur in the regional lymph nodes, and 30% arise at distant sites [8]. Despite the known benefits of early detection of recurrence, no evidence-based surveillance guidelines exist and clinical patterns vary widely [8]. Thus, it is important to define optimal follow-up strategies, including the most effective tests and evaluation intervals. 

The increased number of melanoma survivors poses several potential clinical issues. First, as more cancer patients survive, doctors have an obligation to provide effective posttreatment surveillance as well as evaluate and treat new patients [9–12]. Second, it has been estimated that as many as 72% of melanoma patients detect their own recurrences; thus, it may be argued that patients provide adequate surveillance on their own [13]. However, this estimate may be flawed as studies have not determined whether these recurrences were directly or indirectly detected (direct measures are those in which patients primarily detect their own recurrence; indirect measures are those in which a patient initiates followup with a physician, who subsequently detects the recurrence) [14]. In addition, the actual rate of patient-detected recurrences is unclear because multiple strategies have been used to evaluate recurrence detection [14].

Despite advances in imaging technology, most recurrences are detected on clinical examination by the patient or physician [13]. Whether there is a survival benefit from early detection of metastases is controversial because patients with disseminated disease have historically had limited treatment options [14]. Assuming that early detection of melanoma metastases results is beneficial, identifying the most effective contemporary diagnostic imaging modalities is crucial. We recently performed a meta-analysis of the published medical literature [15] using statistical Bayesian bivariate binomial models to combine the data [16]. 

## 2. Methods

Patient-level data was extracted from published studies which used ultrasonography, computed tomography (CT), positron emission tomography (PET), and CT and PET combined (CT-PET) to detect regional lymph nodes and distant metastases in melanoma patients. The eligibility criterion for study inclusion was a minimum of 6 months of posttreatment followup to provide an opportunity for additional imaging or clinical information to determine the accuracy and precision of these imaging modalities. Imaging results were classified as true positive, true negative, false negative, or false positive using primarily histologic analyses of suspicious sites (lymph node specimens or distant metastases) or clinical outcomes after a minimum of 6 months of postdiagnosis surveillance. A 14-item Quality Assessment of Diagnostic Accuracy Studies (QUADAS) scale was used to evaluate each study to determine potential sources of bias within each of the articles included in the meta-analysis [17]. 

In addition to sensitivity and specificity, the diagnostic odds ratio (calculated as the (TP/FN)/(FP/TN)) was also used as an indicator of test performance. The 95% credible intervals (CrI), which are used in Bayesian statistics for interval estimation, were calculated for each test estimate. Bayesian models were used for the meta-analysis because they can be applied to both large and small studies without ad hoc correction [16]. The positive predictive value for each imaging modality was also calculated for patients with estimated low (5%), intermediate (15%), and high (30%) risks of recurrence.

## 3. Results and Discussion

**Figure 1:**
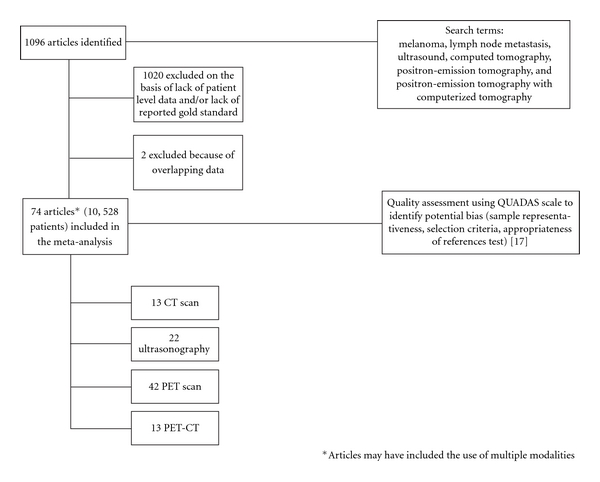
Meta-analysis selection process.

We identified 74 studies that evaluated the utility of ultrasonography, CT, PET, and CT-PET (Figure 1) for the detection of melanoma recurrence. These studies included data from 10,528 melanoma patients, whose data were extracted and used to create two-by-two tables. The mean overall QUAdAS score for all of the studies was 5.8, with a standard deviation of 2.5, indicating that most of the studies were of only fair quality but satisfied the needs for this assessment.

Among the four imaging modalities, ultrasonography had the highest sensitivity, 96% (95% CrI: 85–99); specificity, 99% (95% CrI: 95–100); diagnostic odds ratio, 1675 (95% CrI: 226.6–15920) for lymph node metastasis surveillance compared with CT, PET, and PET-CT (Table 1). Similarly, ultrasonography had the highest positive predictive value, 83% (95% CrI = 36–100), for the detection of lymph node metastasis. For the surveillance of distant melanoma metastasis, PET-CT had the highest sensitivity, 86% (95% CrI: 76–93) and diagnostic odds ratio, 67 (95% CrI: 76–93). 

The results of this meta-analysis indicate that the anatomic site to be evaluated should be considered when choosing an imaging modality. Of the four diagnostic imaging modalities evaluated, ultrasonography was the most sensitive and specific and had the highest positive predictive value for lymph node assessment. PET-CT was the most sensitive and specific and had the highest positive predictive value for distant metastasis surveillance, but it also had the highest number of false positives, resulting in lower specificity and loss of precision. Furthermore, for patients at low risk of metastasis, the low positive predictive value of PET-CT at 33% (95% CrI: 9–61) suggests that its routine use is not warranted in this scenario without additional clinical indications. 

The meta-analysis included all eligible studies from 1990 to 2009 with data on contemporary imaging modalities used for the surveillance of melanoma survivors. The literature search was performed using MEDLINE (from January 1, 1990, through June 30, 2009), Cancerlit (from January 1, 1990, through October 31, 2002), and the Controlled Trials Register from the Cochrane Library (from January 1, 1990, through June 30, 2009) and key words: “melanoma”; “lymph node metastasis”; “ultrasound”; “computed tomography”; “positron-emission tomography”; “positron emission tomography with computerized tomography.” Publications were only eligible if they were in English. An additional strength of the analysis is the Bayesian model which was applied in order to appropriately integrate heterogeneous data from both large and small studies [18]. Limitations of the meta-analysis include the advancement of diagnostic imaging technology over the past ten years as well as potential selection bias of studies included. Laboratory assessment has also been used for surveillance of melanoma recurrences but was not included in the meta-analysis; however, no prospective studies exist to support its use [1]. Such assessments may not be necessary for effective postdiagnosis surveillance.

It is widely acknowledged that no evidence-based strategies exist for most cancers, including melanoma [19]. As the number of cancer survivors and the availability of contemporary medical technologies increase, so does the cost of survivorship care [14]. Over a decade ago, Brobeil et al. [20] reported that recurrence screening in melanoma survivors accounted for an estimated 80% of care costs which could total $27 to $32 million to provide effective surveillance to all patients over a 20-year period.

Creating effective and cost-effective clinical practice guidelines for posttreatment cancer surveillance is critical. Such guidelines, if evidence based, may be useful for measuring quality of care in different settings [21–29]. 

The next step in evaluating the evidence for melanoma practice guidelines is to evaluate the appropriate intervals for followup in patients at varying risk of recurrence [30]. While the gold standard for such an evaluation is a randomized controlled study, such studies with multiple comparisons and long follow-up intervals are difficult to conduct. Melanoma surveillance recommendations should consider type of evaluation (examination with or without diagnostic imaging), frequency, and duration. In their research, Brobeil et al. [20] found that an intensive follow-up program led to a higher detection rate of melanoma in situ (noninvasive stage I melanomas) and a lower detection rate of invasive melanomas, indicating that frequent follow-up examinations may be beneficial. However, the investigators acknowledged that their recommendations were not particularly cost effective, which may lead to decreased implementation across the medical community. Other studies have reported that intense, frequent followups serve only to increase patient anxiety [14]. Thus, it is important to find a balance between intense follow-up schedules and minimal schedules, which may result in early-stage recurrences going undetected.

## Figures and Tables

**Table 1:** Surveillance of melanoma patients (adapted from Xing et al. [15]).

Surveillance imaging modality findings			
Method	Sensitivity	Specificity	Diagnostic odds ratio
Ultrasound	96% (85%–99%)	99% (95%–100%)	1675 (226.6–15920)
CT scan	61% (15%–93%)	97% (70%–100%)	46.25 (2.27–1354)
PET scan	87% (67%–96%)	98% (93%–100%)	391 (68–2737)
CT-PET scan	65% (20%–93%)	99% (92%–100%)	196 (10.77–4675)
